# Beyond weight loss: the value of physical activity and exercise for improving cardiometabolic risk factors in individuals with overweight and obesity

**DOI:** 10.1007/s12471-026-02076-0

**Published:** 2026-09-14

**Authors:** Bente M. de Roos, Valerie M. Monpellier, Dominique Hansen, Thijs M. H. Eijsvogels, Dick H. J. Thijssen, Jan H. M. Karregat

**Affiliations:** 1 Department of Medical Biosciences, Radboud University Medical Center, Nijmegen, The Netherlands; 2 Nederlandse Obesitas Kliniek (Dutch Obesity Clinic), Huis Ter Heide, The Netherlands; 3 Department of Endocrinology & Metabolism, Amsterdam Institute for Gastroenterology, Endocrinology and Metabolism, Amsterdam University Medical Center, Amsterdam, The Netherlands; 4 Faculty of Rehabilitation Sciences, Hasselt University, Hasselt, Belgium

**Keywords:** Overweight, Obesity, Cardiometabolic Risk Factors, Exercise Training, Aerobic Exercise, Resistance Training

## Abstract

The global prevalence of overweight and obesity has increased markedly over recent decades. In clinical obesity care, and in the management of other chronic conditions, the role of physical exercise has largely been evaluated in the context of weight loss. Yet, while it only leads to modest changes in body weight, structured exercise provides extensive health benefits beyond weight loss. A multimodal exercise approach, progressing from reducing sedentary time to incorporating aerobic and resistance training, can provide an effective strategy to improve cardiovascular and metabolic health and body composition. Moreover, integrating structured exercise into surgical and pharmacological obesity care trajectories may support long-term, post-treatment health improvements. This narrative review synthesizes evidence on the effect of exercise on cardiometabolic health and body composition in individuals with overweight and obesity. Furthermore, we also examine the role of structured exercise in supporting cardiometabolic health and preserving skeletal muscle mass during surgical and pharmacological obesity treatment.

## Introduction

Obesity is a progressive and relapsing chronic disease, characterized by an unfavourable body composition with excessive adipose tissue that adversely affects cardiometabolic health [1]. Obesity has emerged as one of the most pressing global health threats, with its prevalence having more than doubled over the past thirty years [2]. Although the management of overweight and obesity was traditionally strongly focused on weight loss [3], improving body composition and cardiometabolic health is ultimately the primary goal of treatment.

Increasing habitual physical activity and structured exercise training are routinely recommended as lifestyle modifications in clinical obesity care to support weight loss. Physical activity encompasses all movement that increases energy expenditure, whereas structured exercise reflects structured and repetitive movement aimed at improving physical fitness (Tab. 1). However, exercise training as a standalone lifestyle intervention generally results in mild-to-modest weight loss, typically < 5% of total body weight [4, 5]. This may be explained by the challenge of achieving a sustained energy deficit through physical activity alone, as well as compensatory physiological and behavioural responses that attenuate the net energy deficit. The overall cardiometabolic benefits, however, go well beyond the exercise-training-associated weight loss. Indeed, emerging evidence indicates that structured exercise training results in substantial improvements in cardiometabolic benefits that occur largely independent of weight loss and are potentially mediated by exercise-induced adaptations in skeletal muscle mass and quality, including enhanced insulin sensitivity, reduced visceral adipose tissue, decreased blood pressure, and enhanced cardiorespiratory fitness [5–7]. Because individuals with overweight and obesity often present with impaired metabolic function, low-grade inflammation, endothelial dysfunction, and increased fat deposition, exercise-induced adaptations may counteract important drivers of cardiometabolic dysregulation, even in the absence of significant weight loss [5–7].

**Table 1:** Exercise terms and definitions [33, 34]

Term	Definition
Physical activity	Any bodily movement produced by skeletal muscles that results in energy expenditure
Exercise	Physical activity that is structured and repetitive and has the aim to maintain or improve physical fitness
*Intensity*	
Low intensity	Physical activity or exercise that is performed between 1.5–3 METs or < 40% HRR (e.g. walking, low-paced cycling, light household chores)
Moderate intensity	Physical activity or exercise that is performed at 3.0–5.9 METs or 40–58% HRR (e.g. brisk walking, moderate cycling, gardening; Moderate-intensity continuous training (MICT) = 30 min continuous moderate intensity activity)
Vigorous intensity	Physical activity or exercise that is performed at ≥ 6.0 METs or 60–89% HRR (e.g. running, fast cycling; High-intensity interval training (HIIT) = repeated short bouts of high intensity activity with brief recovery periods)
*Type*	
Aerobic	Exercise to improve cardiorespiratory fitness (e.g. walking, cycling, swimming, running)
Resistance	Exercise to increase skeletal muscle strength (e.g. weightlifting, resistance band/bodyweight exercises)
Combined	Training programs incorporating both aerobic and resistance exercise

Metabolic Equivalent of Task (*MET*) represents the energy expenditure of an activity relative to the energy expenditure at rest. 1 MET corresponds to the resting oxygen consumption of approximately 3.5 mL O2/kg/min; Heart Rate Reserve (*HRR*) = Maximum Heart Rate − Resting Heart Rate

More recently, surgical (e.g., metabolic bariatric surgery) and pharmacological obesity treatments have proven highly effective for inducing weight loss, with weight reductions of ~20% with pharmacological [8] therapies and up to ~35% following metabolic bariatric surgery [9], driving their rapid uptake in clinical practice. However, some limitations of these weight loss strategies should be mentioned, including skeletal muscle loss [9, 10]. Structured exercise might therefore also play an important role as a complementary strategy for achieving and maintaining long-term improvements in cardiometabolic health and body composition.

**Fig. 1:**
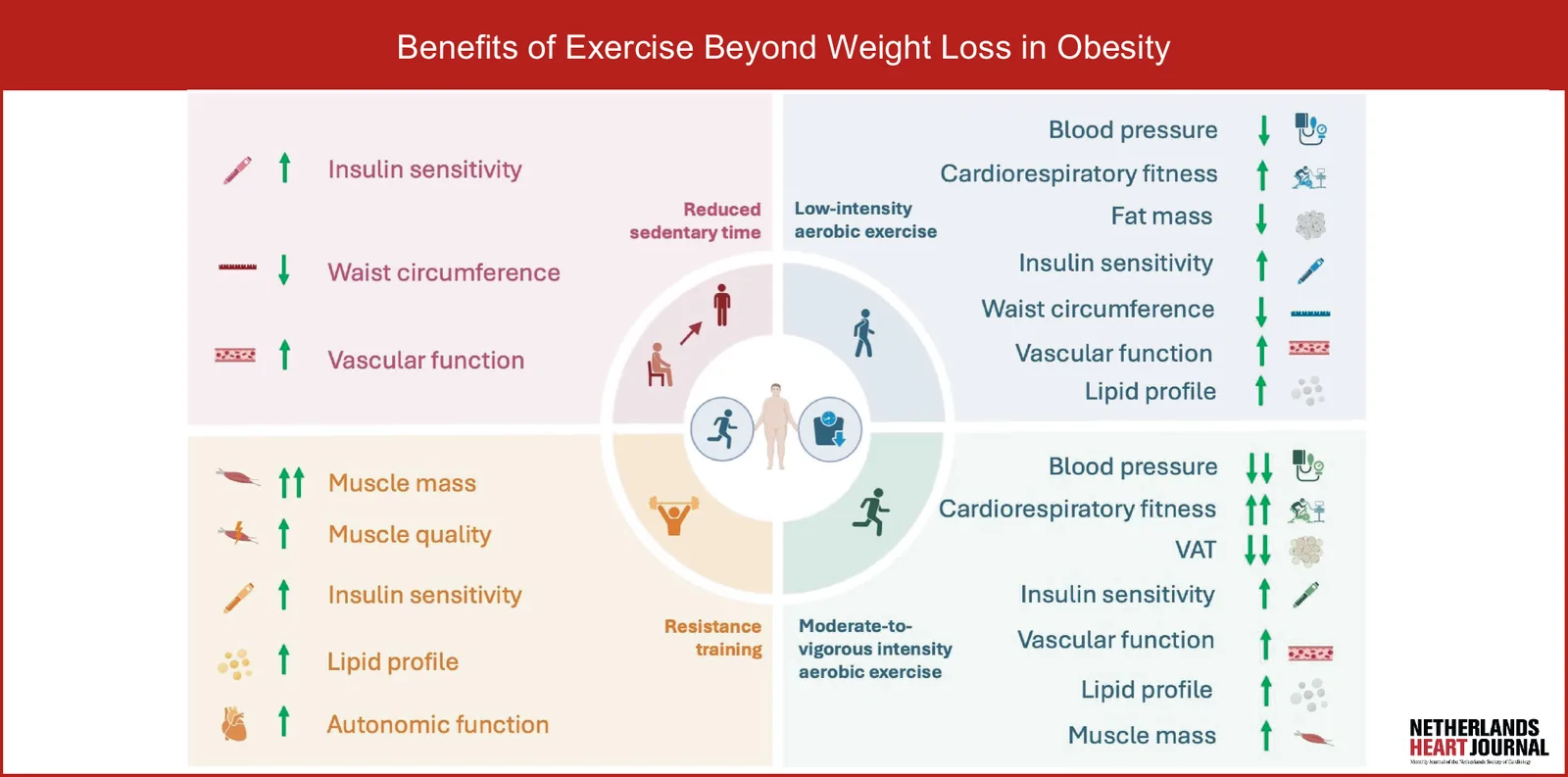
Effects of different modalities of physical activity and exercise on cardiometabolic health outcomes in individuals with obesity

This narrative review synthesizes current evidence on the cardiometabolic benefits of physical activity and structured exercise training, which occur independent of weight loss in individuals with overweight or obesity (Fig. 1) and examines the role of exercise training in optimising the benefits from surgical and pharmacological obesity treatment.

## What are the benefits of exercise on cardiometabolic outcomes in obesity?

In individuals with overweight and obesity, regular exercise training is associated with substantial improvements in cardiovascular function and metabolic regulation that occur independently of changes in body weight [11, 12]. Importantly, changes in body weight or BMI may not adequately reflect exercise-induced metabolic adaptations. For example, visceral adipose tissue is a strong predictor of cardiometabolic risk, and exercise can reduce visceral adipose tissue even in the absence of significant weight loss, suggesting that body weight alone may be a poor marker of exercise-induced benefits [13]. These benefits are of particular clinical significance since overweight and obesity are characterized by impaired cardiovascular function, adverse lipid profiles, autonomic dysregulation, and abnormalities in adipose tissue and skeletal muscle. Importantly, these beneficial adaptations occur largely independently of changes in body weight and are better reflected by changes in body composition and cardiometabolic function. Below we summarize the most important exercise-induced adaptations that contribute to a lower cardiometabolic risk.

### Cardiorespiratory fitness and vascular health

One of the most clinically relevant benefits of exercise is the improvement in cardiorespiratory fitness (VO_2_max), which is one of the strongest predictors of cardiometabolic morbidity and mortality [14]. Even modest increases in VO_2_max are associated with substantial reductions in cardiovascular risk. For example, an improvement of 1 mL/kg/min in VO_2_max is associated with a 2.3% lower risk of all-cause mortality in the general population [15]. In individuals with overweight and obesity, structured exercise training can promote substantial improvements in VO_2_max, reflecting favourable changes in both cardiac output and peripheral oxygen extraction [12].

Hypertension is highly prevalent among individuals with overweight and obesity. In this population, regular structured exercise training leads to substantial reductions in systolic blood pressure of approximately 2–5 mm Hg and in diastolic blood pressure of approximately 1–3 mm Hg, with the largest effects observed in individuals with elevated baseline values [11]. These effects are most likely mediated by vascular adaptations such as reduced arterial stiffness and peripheral resistance, together with improved endothelial function, reflecting an overall healthier vasculature. Moreover, repeated shear stress during exercise enhances endothelial function, stimulates flow-mediated dilatation and increases aerobic capacity [16], most likely through activation of endothelial nitric oxide synthase [17]. The release of nitric oxide induces vasodilatation and reduces platelet aggregation, ultimately contributing a lower risk of atherosclerosis and cardiovascular events [17, 18].

### Autonomic function

Overweight and obesity are associated with autonomic dysregulation, characterised by reduced parasympathetic activity, elevated sympathetic activity, and impaired heart rate variability (HRV), leading to elevated resting heart rate and increased cardiac workload [19]. Structured exercise training has been shown to significantly improve HRV in individuals with obesity, including increased high-frequency (HF) power and reductions in both low-frequency (LF) power and the HF/LF ratio, an index of autonomic balance, indicating a shift toward enhanced parasympathetic activity [20]. These changes indicate a healthier cardiac autonomic balance [20]. Enhanced parasympathetic activity, primarily controlled by the vagus nerve, contributes to a lower resting heart rate, consequently, reduced cardiac workload [19].

### Lipid metabolism

Obesity is associated with adverse lipid profiles, including elevated total cholesterol, triglycerides and low-density lipoprotein (LDL) cholesterol, all of which contribute to increased cardiovascular disease risk [21, 22]. Structured exercise training can induce favourable changes in lipid profiles in individuals with overweight and obesity. Exercise interventions have been shown to effectively reduce LDL cholesterol concentrations and increase high-density lipoprotein (HDL) cholesterol concentrations [23]. These changes seem driven by increased exercise-induced energy expenditure, enhancing lipoprotein lipase activity and post-exercise fat oxidation [23]. Together, these changes may further contribute to improvements in vascular health and attenuating of the progression of atherosclerosis, subsequently contributing to a reduction in overall cardiometabolic risk [22].

### Insulin sensitivity

Insulin resistance is a frequently observed metabolic impairment in individuals with overweight and obesity and is a key mechanism underlying the development of type 2 diabetes mellitus. Alterations in skeletal muscle, characterized by increased intramuscular fat infiltration and impaired mitochondrial function, are important contributors to insulin resistance [24]. Structured exercise enhances insulin sensitivity, through increased insulin-mediated glucose uptake and improved mitochondrial efficiency [25]. By improving muscle quality and increasing skeletal muscle mass, exercise training further supports glucose uptake through both insulin-mediated and insulin-independent pathways, including enhanced glucose transporter 4 (GLUT4) expression and translocation, resulting in improved whole-body glucose homeostasis [24].

## What are the benefits of exercise training on body composition in obesity?

### Adipose tissue

Individuals with overweight and obesity generally exhibit adipose tissue dysfunction, characterised by excess visceral adipose tissue (VAT), impaired subcutaneous adipose tissue (SCAT) storage capacity, and increased secretion of pro-inflammatory adipokines (e.g., leptin, TNF‑α, IL-6) [26]. Subcutaneous adipose tissue dysfunction reflects a reduced capacity to store excess lipids, leading to fat accumulation. Visceral adipose tissue, located primarily in the mesentery and omentum, is considered the most metabolically harmful fat depot. Visceral adipose tissue is known to be highly vascularised and contains larger and more insulin-resistant adipocytes, exhibiting hyper-lipolytic characteristics [26]. These characteristics contribute to increased secretion of pro-inflammatory adipokines and cytokines [27], which play a key role in the development of cardiometabolic diseases [28]. In contrast, subcutaneous adipose tissue, located primarily in the femoral and gluteal regions, is characterised by a larger number of smaller adipocytes that are more insulin sensitive and act as a metabolic buffer by storing triglycerides [26]. However, in individuals with overweight and obesity, the storage capacity of subcutaneous adipose tissue can become dysregulated, causing lipid overflow into visceral adipose tissue and around the organs [26].

Structured exercise induces structural and functional adaptations within adipose tissue, counteracting these abnormalities, even in the absence of significant weight loss [13]. A large network meta-analysis showed that both moderate- and vigorous-intensity aerobic exercise induce meaningful reductions in visceral adipose tissue and subcutaneous adipose tissue volumes [29]. These structural adaptations are associated with improved insulin sensitivity and reduced secretion of inflammatory adipokines and cytokines, thereby lowering cardiometabolic risk [30].

### Skeletal muscle tissue

In individuals with overweight and obesity, skeletal muscle mass often exhibits structural and functional abnormalities, including reduced muscle strength relative to body weight and increased intermuscular adipose tissue, which can contribute to impairments in mobility [25, 31]. As adipose tissue accumulates within and between muscle fibers, mitochondrial function and contractile properties decline, contributing to insulin resistance and reduced muscle strength and function relative to muscle size [25]. In individuals with overweight and obesity, this muscle phenotype contributes to the risk of developing sarcopenic obesity, which is characterised by relatively low skeletal muscle mass in proportion to total body weight combined with reduced muscle function, increasing the risk of physical limitations and metabolic disease [25]. Structured exercise training counteracts these impairments by improving muscle density and lowering intramuscular fat infiltration, thereby improving insulin sensitivity and skeletal muscle metabolism [25]. In addition to these structural changes, exercise has also been shown to enhance muscle quality, defined as the amount of muscle strength generated relative to muscle size [32], contributing to improved physical functioning and reducing the risk of sarcopenic obesity.

## Effects of different PA modalities

The physical activity exists on a continuum ranging from sedentary behaviour to vigorous-intensity exercise (Tab. 1), with each modality being associated with distinct physiological adaptations.

### Reducing sedentary time

Reducing sedentary time offers an accessible strategy for individuals who face barriers to structured exercise, modestly improving vascular function and metabolic regulation [35]. In individuals with overweight and obesity, replacing prolonged sitting with light-intensity physical activity improves triglyceride and HDL-cholesterol levels; reduces waist circumference, fasting insulin, and all-cause mortality risk; and reduces E‑selectin concentrations (i.e., a biomarker for endothelial activation, inflammation, and cardiovascular disease) [35]. These changes indicate early vascular and metabolic adaptations that contribute to improved endothelial function and reduced low-grade inflammation. Decreased sedentary time also enhances glycaemic control by increasing non-exercise activity thermogenesis and improved insulin sensitivity through increased skeletal muscle activity [36]. Interruption of sedentary time enhances contractile activity in skeletal muscle, increasing glucose uptake and mitochondrial function [37].

### Low-intensity aerobic exercise

Low-intensity aerobic exercise training (Tab. 1) can induce modest but clinically relevant improvements in body composition, functional performance, and metabolic health. Six weeks of low-intensity exercise training (30–39% of HR reserve, calculated as the difference between maximal and resting HR) has been associated with significant reductions in body mass index (BMI), waist circumference, and total body fat as well as improvements in functional capacity [38]. Low-intensity exercise training has also been associated with favourable metabolic changes, including decreased LDL cholesterol, increased HDL cholesterol, and improved insulin sensitivity [38], and six months of low-to-moderate intensity exercise training has been linked to reduced HbA1c and LDL cholesterol and increased cardiorespiratory fitness [39]. These effects are likely mediated by repeated muscle activation, increasing oxidative metabolism and enhancing insulin-dependent glucose uptake [39].

### Moderate-to-vigorous aerobic exercise

Moderate-to-vigorous aerobic exercise induces the most pronounced and wide-ranging cardiometabolic benefits in individuals with overweight and obesity. Both moderate-intensity continuous training (MICT) and high-intensity interval training (HIIT) substantially improve cardiorespiratory fitness [40] across different age groups and baseline fitness levels [40–42]. MICT and HIIT effectively reduce systolic and diastolic blood pressure, particularly in individuals with elevated baseline values [11], with the most pronounced improvements observed following HIIT [42]. These reductions most likely relate to shear-stress-mediated enhancements in vascular and endothelial function, remodelling of arterioles and/or changes in central regulation of blood pressure (e.g., baroreflex sensitivity). Moderate-to-vigorous aerobic exercise also reduces visceral adipose tissue, even in the absence of substantial weight loss [43]. These changes are strongly associated with improved insulin sensitivity and reduced chronic inflammation [27]. MICT and HIIT also induce favourable body composition changes, including reductions in adipocyte size, improved insulin-mediated inhibition of lipolysis [44] and increased proportions of type I muscle fibres with high oxidative capacity [45], with more pronounced effects following longer-duration aerobic interventions [46].

### Resistance training

Individuals with overweight and obesity often experience lower relative muscle strength, infiltration of inter- and intramuscular fat, and impaired neuromuscular performance [32], which contribute to limited functional capacity and impaired metabolic health. Resistance training (RT) effectively addresses these impairments by stimulating muscle protein synthesis, increasing skeletal muscle mass, and improving skeletal muscle quality [25, 32]. An 18-month diet-induced weight loss intervention combined with either aerobic exercise training or RT showed that RT was significantly more effective in preventing skeletal muscle mass loss, reducing intramuscular fat, and inducing a more favourable muscle-to-fat loss ratio [25]. While aerobic exercise tends to induce a muscle fibre switch towards the slow-twitch type I muscle fibres [45], a sixteen-week RT intervention has been shown to increase the proportion and function of fast-twitch type II muscle fibres [47, 48], which are important for acute high-force production, functional capacity and glycaemic control [49]. In addition, RT has been shown to improve HRV by reducing sympathetic activation and increasing parasympathetic activity, and promote favourable changes in blood pressure and lipid profiles [50].

### Combined modalities

Combined aerobic exercise training and RT is often recommended for individuals with overweight and obesity, as these modalities target different but complementary physiological systems [7]. Evidence suggests that this multimodal approach can yield broader cardiometabolic adaptations than either modality alone. For example, combined training has been shown to produce larger reductions in blood pressure and body fat and larger improvements in glycaemic control, while simultaneously improving cardiorespiratory fitness and muscular strength [51]. However, other comparative trials have reported no additional cardiometabolic benefit of combined training [12, 52, 53]. In addition, the evidence base for combined modalities remains substantially smaller and less consistent compared to that for single-modality interventions. Given this relatively limited and heterogeneous evidence base, definitive conclusions regarding the superiority of combined modalities over single modalities cannot yet be drawn. Nevertheless, the indications for broader and potentially greater cardiometabolic benefits support further evaluation of combined interventions in larger, methodologically consistent RCTs.

## What are the effects of combining physical activity and exercise training with surgical or pharmacological obesity treatment?

**Fig. 2:**
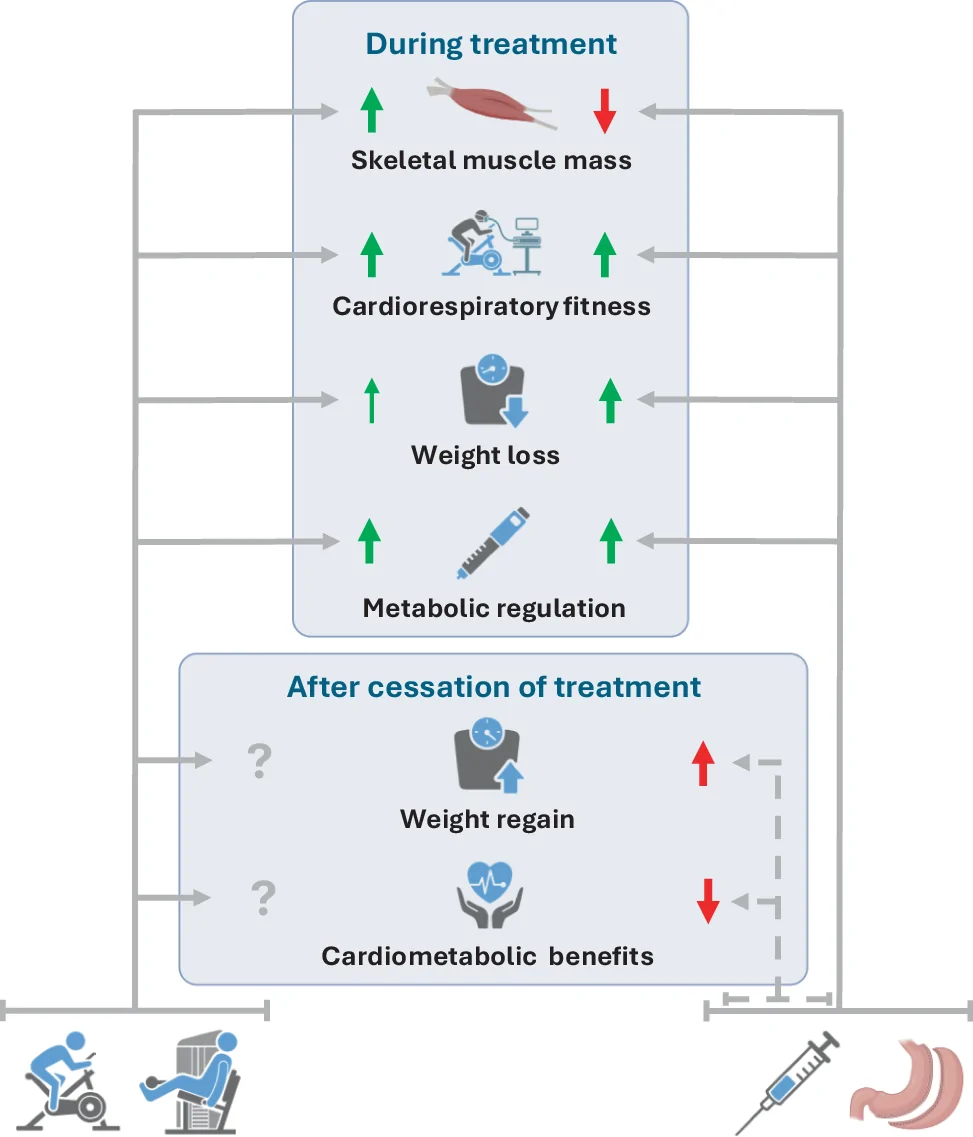
Schematic overview of the effects of obesity treatment and physical activity & exercise training on bodyweight, metabolic regulation, cardiorespiratory fitness, and skeletal muscle mass

Pharmacological and surgical treatments are increasingly used in the management of obesity and have demonstrated remarkable efficacy, with weight reductions of up to 20% [8] and 35% [9] of total body weight, respectively. This magnitude of weight loss is associated with substantial improvements in cardiometabolic risk factors, including blood pressure, lipid profiles, and glycaemic regulation [54]. Despite these benefits, both surgical and pharmacological interventions are associated with significant reductions in lean body mass [9, 10], largely reflecting reductions in metabolically active skeletal muscle mass. In older individuals (≥ 45 years) undergoing metabolic bariatric surgery, disproportionate loss of lean body mass has been associated with an increased risk of cardiovascular events during follow-up [55]. These observations highlight the importance of monitoring and preserving skeletal muscle mass and function during anti-obesity treatment, particularly because exercise training yields additional benefits on cardiometabolic health that extend beyond weight loss alone. Figure 2 provides a schematic overview of the effects of obesity treatment on body weight and cardiometabolic outcomes, together with the hypothesized contribution of physical activity and exercise training.

RT is the most effective strategy for inducing skeletal muscle hypertrophy and increasing muscle strength [56]. Structured moderate- to high-load whole-body RT, particularly when combined with adequate dietary protein intake, has been proposed to help preserve skeletal muscle mass during obesity treatment [57]. However, evidence supporting this approach in the context of surgical or pharmacological obesity treatment remains limited. A recent meta-analysis in patients undergoing metabolic bariatric surgery, synthesizing interventions ranging from 1 to 24 months, showed that RT-based exercise interventions effectively increased muscle strength, but resulted in only a modest attenuation of lean body mass loss, with a pooled, non-significant mean difference of approximately +0.7 kg compared to control conditions [58].

Beyond lean mass loss, the maintenance of weight loss following pharmacological treatment is highly dependent on continued medication exposure, with treatment discontinuation often resulting in partial or complete weight regain [54]. These observations suggest that pharmacotherapy-induced cardiometabolic improvements may be partially reversible and highlight the potential role of exercise training in promoting more durable physiological adaptations, although evidence on the long-term efficacy of exercise-induced adaptations during pharmacotherapy remains limited. Integrating structured exercise training into obesity treatment strategies may optimize the benefits of both surgical and pharmacological interventions by mitigating skeletal muscle loss, enhancing cardiometabolic health, and supporting sustainable long-term weight management. Nevertheless, relatively few studies have previously evaluated such integrated exercise-based approaches in individuals undergoing surgical or pharmacological obesity treatment, underscoring the need for more research evaluating the efficacy and feasibility of structured exercise training in these specific populations.

## Clinical implications

Physical activity and exercise training play a central role in the management of overweight and obesity. Regular physical activity improves multiple cardiometabolic and functional outcomes, even in the absence of substantial weight loss. These broad effects underscore the clinical importance of physical activity and exercise, given that cardiovascular risk management aims to reduce overall cardiometabolic risk, rather than to target individual risk factors in isolation.

Reducing sedentary time is a first step to improve health in individuals with overweight and obesity, particularly in those with a low functional capacity or barriers to structured exercise. Reducing sedentary time should target both the volume and the pattern of sedentary time. Sedentary time exceeding ~ 9.5 h/day is associated with increased mortality risk, while interrupting prolonged sitting (e.g., every ~ 30 min) may provide additional benefits for cardiometabolic health [59].

Reducing sedentary time enhances vascular function and metabolic regulation. As functional capacity improves, structured low-intensity exercise can be introduced to induce more substantial improvements in body composition, functional capacity, and metabolic health.

More robust evidence supports the use of moderate-to-vigorous aerobic exercise to achieve larger improvements in cardiorespiratory fitness, visceral adipose tissue reduction, blood pressure regulation, and systemic inflammation. These improvements are strongly linked to reductions in long-term cardiovascular and metabolic risks. Simultaneously, RT is essential for preserving and improving muscle mass, muscle quality, and neuromuscular function. Combining aerobic exercise and resistance training has the potential to simultaneously target cardiovascular, metabolic, and musculoskeletal pathways, inducing the broadest and most clinically meaningful improvements compared to single-modality approaches. However, evidence suggests that these combined approaches do not consistently yield larger effects compared to single-modality interventions.

Patient communication should emphasize that the broad health benefits of physical activity and exercise go well beyond weight loss alone and may even occur in the absence of weight loss. These benefits range from improving cardiometabolic risk factors (e.g., blood pressure, lipid profile, insulin sensitivity), body composition (e.g., preservation or gain of skeletal muscle mass and reduction in visceral adipose tissue), and physical functioning and strength. Helping patients understand that substantial cardiometabolic health and physical function benefits can be achieved independently of weight loss may enhance motivation and sustained participation in physical activity and exercise. Safety considerations should also be taken into account when prescribing physical activity and exercise. Individuals with overweight and obesity, particularly those who are physically inactive, may have a higher baseline risk of musculoskeletal injury. However, current evidence indicates that participation in structured exercise programs does not increase the risk of injury or illness compared with non-exercising individuals [60]. Accordingly, treatment strategies to optimize cardiometabolic benefits in individuals with overweight and obesity should focus on progressively integrating reduced sedentary time, increasing low-intensity exercise, moderate-to vigorous aerobic exercise, and RT, according to each patient’s capacity, preferences, and goals. In addition, the evaluation of treatment response should extend beyond body weight alone. Parameters such as body composition and measures of physical performance and muscle strength provide a more comprehensive and clinically meaningful assessment of health improvements.

## Conclusion

Physical activity and exercise training provide substantial benefits for individuals with overweight and obesity that extend far beyond weight loss, including reductions in cardiometabolic risk and physical functioning. The evidence summarized in this review suggests that a multimodal and progressively structured exercise approach offers important clinical benefits in individuals with overweight and obesity, reinforcing the role of physical activity and exercise training as fundamental components of comprehensive obesity care in combination with existing obesity treatments.

## Data Availability

All information supporting the findings of this work are included in the article and in the cited references.
